# Curators of Med. Department of University of Buffalo

**Published:** 1852-10

**Authors:** 


*Curators of Med. Department of University of Buffalo.*— The name of Dr. Morgan G. Lewis, of Black Rock, Erie Co., was inadvertently omitted in the annual announcement for this year.

